# Association Between Repeated Prophylactic Antimicrobial Eye Drop Use and Reduced Antimicrobial Susceptibility in Conjunctival and Nasal Commensal Bacteria

**DOI:** 10.3390/antibiotics15080744

**Published:** 2026-07-31

**Authors:** Ryo Tomemori, Kaoru Araki-Sasaki, Tatsuya Kanagawa, Masayuki Ohnaka, Junzo Hisatsune, Koichiro Higasa, Hisanori Imai

**Affiliations:** 1 Department of Ophthalmology, Kansai Medical University, 2-5-1 Shinmachi, Hirakata 5731010, Osaka, Japan; tomemori.ryo@kmu.ac.jp (R.T.); ohnaka.mas@kmu.ac.jp (M.O.); imai.his@kmu.ac.jp (H.I.); 2 Department of iPS Stem Cell Regenerative Medicine, Kansai Medical University, 2-5-1 Shinmachi, Hirakata 5731010, Osaka, Japan; tatsuya.orthptist@gmail.com; 3 Antimicrobial Resistance Research Center, National Institute of Infectious Diseases, Japan Institute for Health Security, Aoba-cho 4-2-1, Higashimurayama 1890002, Tokyo, Japan; hisatsune.j@jihs.go.jp; 4 Department of Genome Analysis, Institute of Biomedical Science, Kansai Medical University, Hirakata 5731010, Osaka, Japan; higasa.koi@kmu.ac.jp

**Keywords:** antimicrobial resistance, quinolone resistance-determining region, *Staphylococcus epidermidis*, conjunctival microbiota, nasal microbiota, topical antimicrobial eye drops

## Abstract

**Background/Objectives:** The objective of this study was to clarify changes in the antimicrobial susceptibility of commensal bacteria in the conjunctival sac and nasal cavity associated with repeated use of prophylactic antimicrobial eye drops during intravitreal anti-vascular endothelial growth factor injections. **Methods:** Patients were divided into Group A, comprising 21 patients before the first use of prophylactic antimicrobial eye drops, and Group B, comprising 26 patients after receiving 20 or more injections. Minimum inhibitory concentration and susceptibility testing were performed on bacteria isolated from the conjunctival sac and nasal cavity using cefmenoxime, ceftazidime, levofloxacin, gatifloxacin, and moxifloxacin. Genetic mutations in the quinolone resistance-determining region of *Staphylococcus epidermidis*, phylogenetic relationships, and the nasal microbiome were also analyzed. **Results:** *Corynebacterium*, *S. epidermidis*, and *Cutibacterium acnes* were detected in both sites. In Group B, *S. epidermidis* and *Corynebacterium* showed reduced susceptibility to quinolones, whereas *C. acnes* did not. GyrA_S84 and ParC_S80 mutation rates in *S. epidermidis* were higher in Group B than in Group A. Among the patients with quinolone-resistant S. epidermidis isolated from both sites, 4 out of 10 patients had paired conjunctival and nasal isolates with the same ST type, suggesting that isolates from the two anatomical sites may be genetically related. The nasal microbiome α- and β-diversity did not differ significantly between groups, although exploratory LEfSe analysis suggested candidate taxon-level differences. **Conclusions:** Repeated prophylactic use of antibiotic eye drops was associated with reduced susceptibility in both the conjunctival sac and nasal cavity.

## 1. Introduction

Measures are being implemented worldwide to address inappropriate antimicrobial use because it contributes to increasing bacterial resistance. However, although national antimicrobial resistance action plans restrict the use of oral and intravenous antimicrobial agents, they do not address antimicrobial eye drops.

Antimicrobial eye drops deliver antimicrobial agents to the eye at concentrations far higher than those achievable through oral or intravenous administration; therefore, the consequences of inappropriate use can be serious.

In ophthalmology, antimicrobial eye drops are widely used for pre- and postoperative prophylaxis, and quinolone eye drops are used particularly frequently in Japan. For example, a 14-day course of gatifloxacin eye drops has been reported to disrupt the microbial flora of the conjunctival sac [1], and decreased microbial susceptibility has also been reported [2].

In recent years, the use of intravitreal injections of anti-VEGF agents for age-related macular degeneration has increased annually, and prophylactic antibiotics are being used during the period before and after injection. According to a 2019 report by Sugimoto et al., quinolone eye drops were used in approximately 72.7% of cases for prophylactic administration prior to intravitreal injections of anti-vascular endothelial growth factor (anti-VEGF) antibodies in Japan [3]. Furthermore, several reports have pointed out that this practice leads to an increase in the proportion of resistant bacteria [4,5,6,7,8]. Intravitreal injections for age-related macular degeneration can be repeated indefinitely. Although previous studies have examined the effects of up to seven cycles, in current clinical practice, patients may undergo many more cycles and repeated antibiotic exposure; therefore, further studies involving a larger number of treatment cycles are needed.

In this study, we investigated the effects of pre- and postoperative administration of antimicrobial eye drops on the bacterial flora of the conjunctival sac and nasal cavity in patients who had undergone more than 20 cycles of anti-VEGF intravitreal injections. Among the isolated bacteria, we previously reported reduced susceptibility of *S. epidermidis* to quinolone antibiotics [9]. In this follow-up study, we report changes in antimicrobial susceptibility in *Corynebacterium* spp. and *Cutibacterium acnes* (*C. acnes*) isolated from the conjunctival sac and nasal cavity. We also examined changes in the nasal microbiome after repeated administration of antimicrobial eye drops. Furthermore, to confirm the association of bacteria between the conjunctival sac and nasal cavity, we assessed the mutation rate of quinolone resistance genes in isolated *S. epidermidis* and performed phylogenetic analysis.

## 2. Results

### 2.1. Bacterial Species Detected in Nasal and Conjunctival Samples

In Group B, the number of repeated intravitreal anti-VEGF injections ranged from 21 to 96 (mean, 43.6 ± 18.6), and prophylactic moxifloxacin eye drops were administered repeatedly in conjunction with each injection cycle. Specimen collection was performed at a median of 50 days (range, 19–264 days) after the final administration of moxifloxacin. *S. epidermidis*, *Corynebacterium*, and *C. acnes* were the most frequently isolated microorganisms in both the conjunctival sac and the nasal cavity. A total of 72 bacterial strains were detected from the conjunctival sac across both groups: *C. acnes* (Group A, 9 strains; Group B, 12 strains), *Corynebacterium* spp. (Group A, 7 strains; Group B, 14 strains), and *S. epidermidis* (Group A, 5 strains; Group B, 10 strains). In the nasal cavity, a total of 156 bacterial strains were detected, distributed as follows: *S. epidermidis* (Group A, 22 strains; Group B, 27 strains), *Corynebacterium* spp. (Group A, 17 strains; Group B, 22 strains), and *C. acnes* (Group A, 7 strains; Group B, 17 strains). The number of isolates represents bacterial strains recovered from culture. We adopted a two-layered analytical approach. The primary analysis was conducted at the isolate level to capture the overall microbiological burden and susceptibility profiles in the study population. A sensitivity analysis restricted to one isolate per patient per species (the first isolate) was performed. Patient-level and isolate-level denominators are provided in Supplementary Table S2.

### 2.2. Minimum Inhibitory Concentrations (MICs) and Susceptibility of Various Antimicrobial Agents

**Figure 1:**
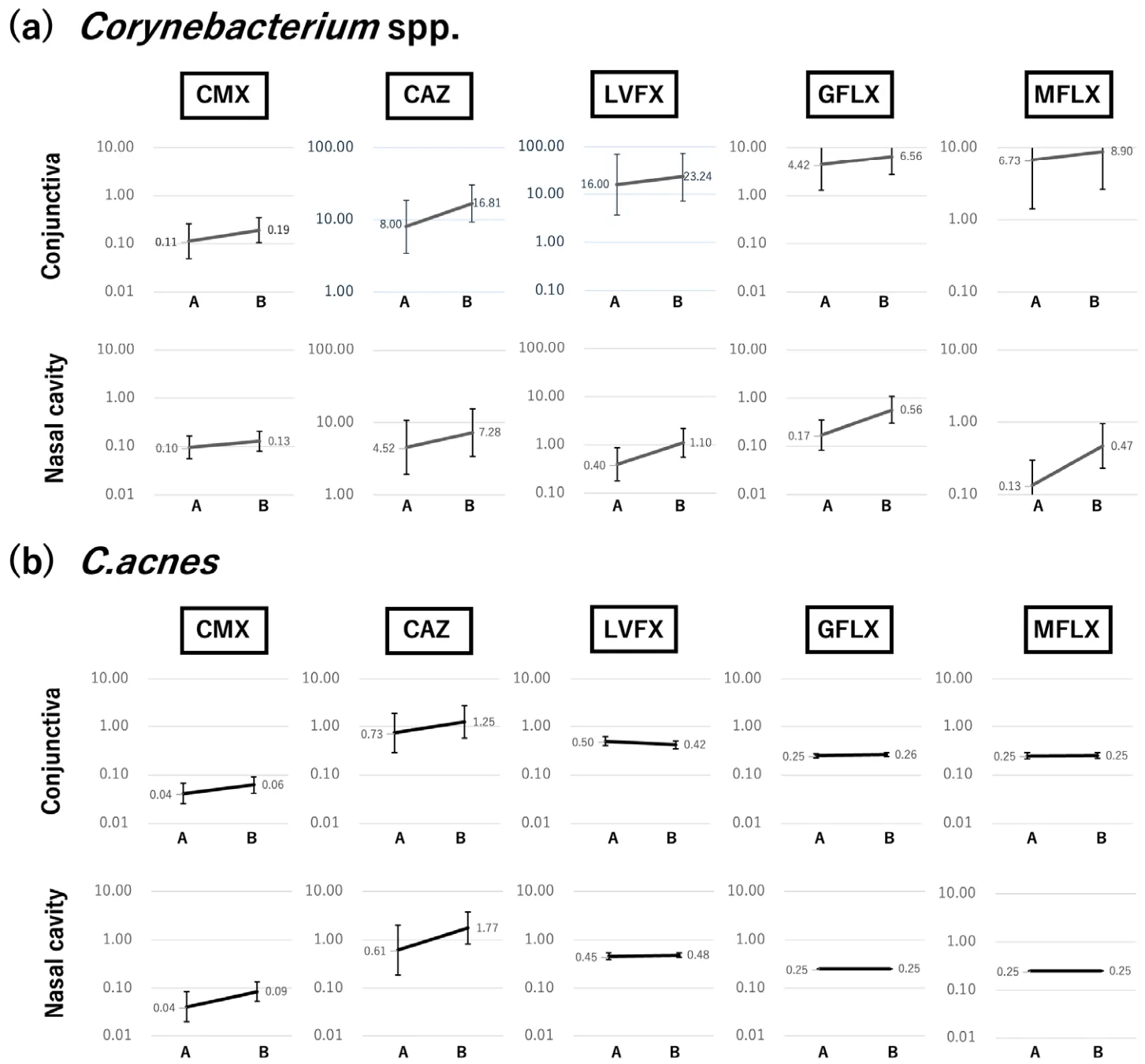
Changes in the mean MIC value (µg/mL) of various antibiotics against *Corynebacterium* spp. (**a**) and *Cutibacterium acnes* (**b**). *Corynebacterium* spp. (**a**) showed higher mean MIC values in Group B than in Group A for all antimicrobial agents tested, especially in the nasal cavity. In particular, the mean MIC values of fluoroquinolones (LVFX, GFLX, and MFLX) were higher in Group B than in Group A. In contrast, for *C. acnes* (**b**), although a slight increase in the mean MIC value of cephalosporin antibiotics was observed in both the conjunctival sac and the nasal cavity, no increase in the mean MIC values of quinolone antibiotics was observed.

Figure 1a,b show the mean MIC values of various antibiotics—cefmenoxime (CMX), ceftazidime (CAZ), levofloxacin (LVFX), gatifloxacin (GFLX), and moxifloxacin (MFLX)—against *Corynebacterium* spp. and *C. acnes* isolated from the conjunctival sac and nasal cavity.

#### 2.2.1. Results for Corynebacterium MIC (Minimum Inhibitory Concentration)

##### Trends in MIC for Conjunctival Isolates

The MICs of the three quinolone agents against conjunctival *Corynebacterium* spp. isolates were generally high, even in Group A. The model-based mean MIC for LVFX increased from 16.00 μg/mL in Group A to 23.24 μg/mL in Group B, representing a 1.45-fold increase. For GFLX, the model-based mean MIC increased from 4.42 to 6.56 μg/mL (1.49-fold), whereas the mean MIC for MFLX increased from 6.73 to 8.90 μg/mL (1.32-fold). However, none of these differences reached statistical significance.

##### Trends in MIC for Nasal Isolates

Among nasal *Corynebacterium* spp. isolates, MICs for all three quinolones remained low in Group A but tended to increase in Group B, although the differences were not statistically significant. The model-based mean MIC for LVFX increased from 0.40 to 1.10 μg/mL, corresponding to a 2.79-fold increase. Similarly, the mean MIC for GFLX increased from 0.17 to 0.56 μg/mL (3.32-fold), and that for MFLX increased from 0.13 to 0.47 μg/mL (3.48-fold).

Changes in the mean MIC values against *S. epidermidis* (isolate-level) are presented in Supplementary Material Figure S1 and Table S1 from our previously published paper [9]. Similarly to *S. epidermidis* [Figure S1], the mean MIC values of cephalosporins and quinolones against *Corynebacterium* spp. were higher in Group B than in Group A (Figure 1a).

#### 2.2.2. Results of *Corynebacterium* Susceptibility Rates

##### Trends in Susceptibility Rates of Conjunctival Isolates (Table 1a–c Upper Panel)

The susceptibility rates of conjunctival *Corynebacterium* spp. isolates to the three quinolones tended to decrease in Group B. For LVFX, the susceptibility rate decreased from 25.0% (2/8 isolates) in Group A to 7.7% (1/13 isolates) in Group B. For GFLX, the susceptibility rates were 30.0% (3/8 isolates) and 23.1% (3/13isolates) in Groups A and B, respectively. For MFLX, the susceptibility rates were 25.0% (2/8 isolates) in Group A and 23.1% (3/13 isolates) in Group B. However, none of these differences were statistically significant.

##### Trends in Susceptibility Rates for Nasal Isolates (Table 1a–c Lower Panel)

Although no statistically significant differences were observed, nasal *Corynebacterium* spp. isolates showed a tendency toward lower susceptibility rates in Group B for all three quinolones. For LVFX, the susceptibility rate decreased from 88.2% (15/17 isolates) in Group A to 59.1% (13/22 isolates) in Group B, corresponding to an absolute difference of 29.1 percentage points. For GFLX, the susceptibility rate decreased from 100.0% (17/17 isolates) in Group A to 81.8% (18/22 isolates) in Group B, representing a difference of 18.2 percentage points. A similar pattern was observed for MFLX, with susceptibility rates decreasing from 100.0% (17/17 isolates) in Group A to 81.8% (18/22 isolates) in Group B.

**Table 1:** Changes in the susceptibility of *Corynebacterium* spp. to various quinolones, LVFX (**a**), GFLX (**b**), and MFLX (**c**), following perioperative administration of antimicrobial eye drops.

(**a**)					
Conjunctival isolates for LVFX	Susceptibility (strains)		Susceptibility rate (%)	95% Confidence Interval	
	+	−		lower limit	upper limit
Group A	2	6	25.0	3.2	65.1
Group B	1	12	7.7	0.2	36.0
Differences between Group A and B	*p* = 0.5308		17.3	−16.0	50.0
Nasal cavity isolates for LVFX	Susceptibility (strains)		Susceptibility rate (%)	95% Confidence Interval	
	+	−		lower limit	upper limit
Group A	15	2	88.2	63.6	98.5
Group B	13	9	59.1	36.4	79.3
Differences between Group A and B	*p* = 0.0733		29.1	3.5	54.8
(**b**)					
Conjunctival isolates for GFLX	Susceptibility (strains)		Susceptibility rate (%)	95% Confidence Interval	
	+	−		lower limit	upper limit
Group A	3	5	30.0	3.7	71.0
Group B	3	10	23.1	4.7	50.8
Differences between Group A and B	*p* = 1.0000		6.9	−32.6	46.9
Nasal cavity isolates for GFLX	Susceptibility (strains)		Susceptibility rate (%)	95% Confidence Interval	
	+	−		lower limit	upper limit
Group A	17	0	100.0	80.5	100.0
Group B	18	4	81.8	59.7	94.8
Differences between Group A and B	*p* = 0.1179		18.2	2.1	34.3
(**c**)					
Conjunctival isolates for MFLX	Susceptibility (strains)		Susceptibility rate (%)	95% Confidence Interval	
	+	−		lower limit	upper limit
Group A	2	6	25.0	3.2	65.1
Group B	3	10	23.1	5.0	53.8
Differences between Group A and B	*p* = 1.0000		1.9	−35.8	39.7
Nasal cavity isolates for MFLX	Susceptibility (strains)		Susceptibility rate (%)	95% Confidence Interval	
	+	−		lower limit	upper limit
Group A	17	0	100.0	80.5	100
Group B	18	4	81.8	59.7	94.8
Differences between Group A and B	*p* = 0.1179		18.2	2.1	34.3

*Corynebacterium* spp. demonstrated reduced susceptibility in Group B, with the most significant differences among nasal isolates. Comparisons of conjunctival isolates had wide confidence intervals that included zero and should be treated with caution.

In the patient-level analysis, the overall patterns of MICs and antimicrobial susceptibility were consistent with those observed in the primary (isolate-level) analysis (Table S2).

In contrast, no significant increase in quinolone MICs was observed for *C. acnes* in either anatomical site in Group B/A. The MIC ratios were close to 1.0 for LVFX, GFLX, and MFLX, suggesting no clear decrease in quinolone susceptibility.

### 2.3. Changes in the Nasal Microbiome

**Figure 2:**
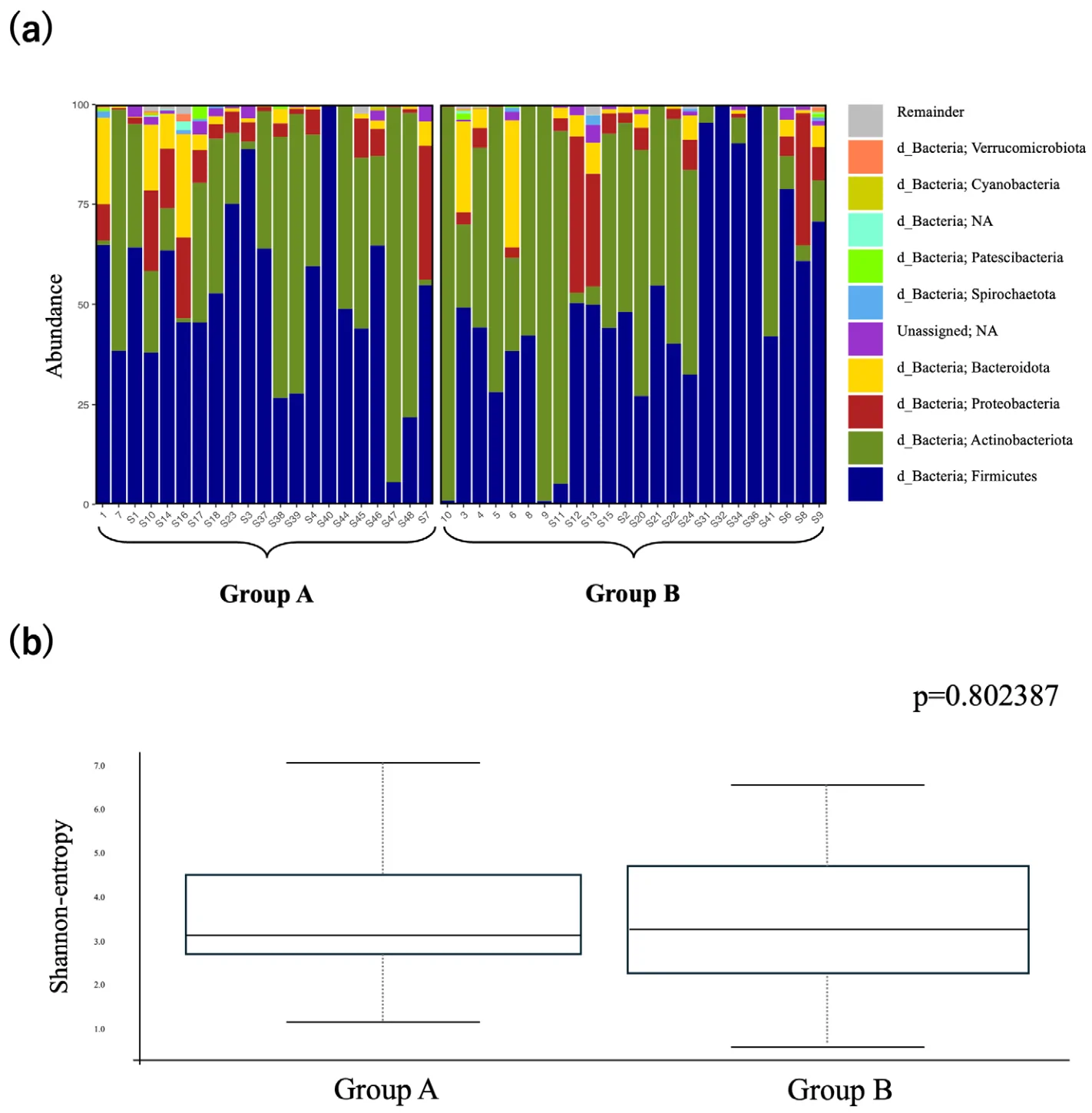

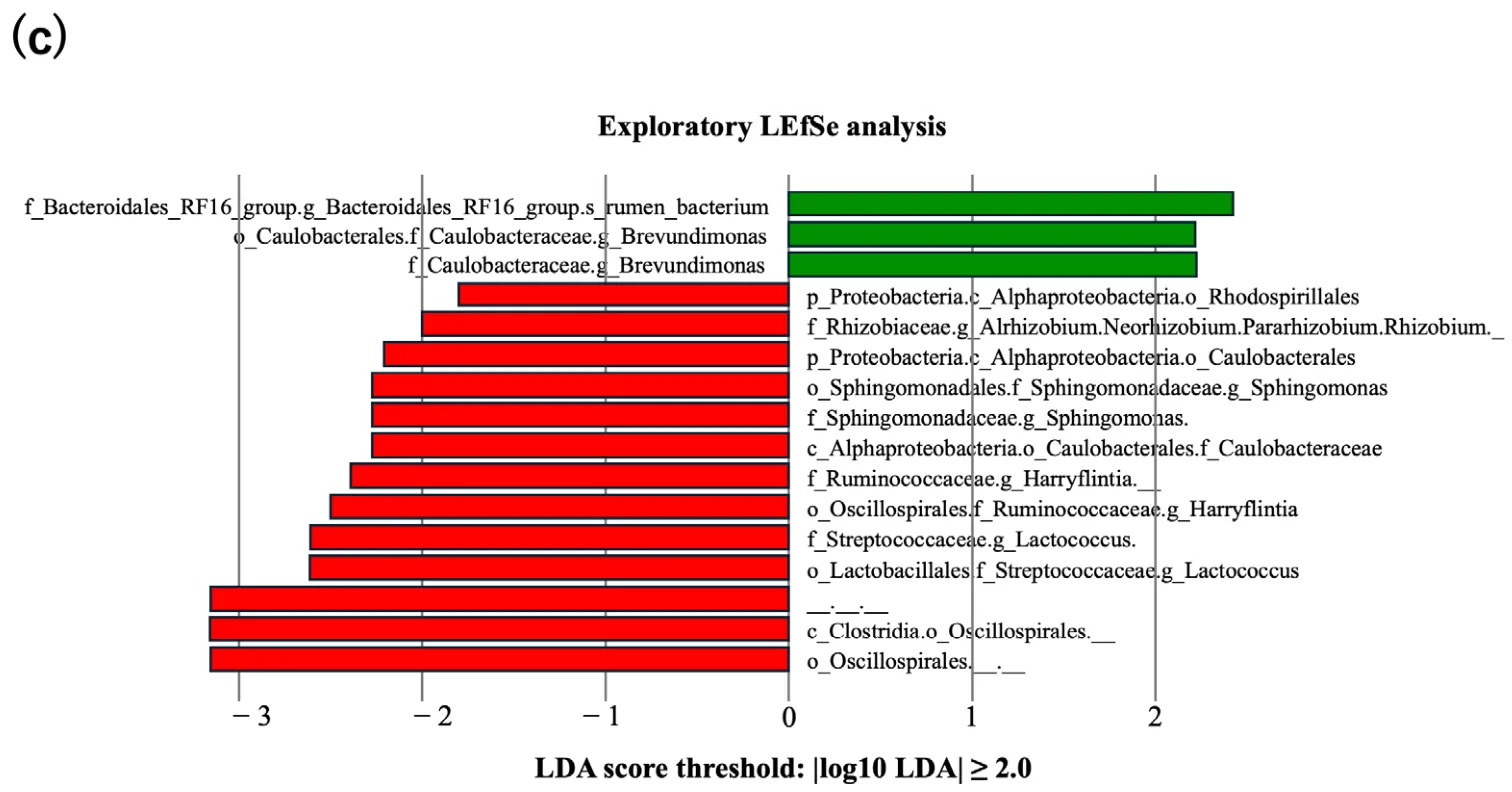
Nasal microbiome composition and diversity in Groups A and B. (**a**) Relative bacterial composition of nasal samples at the phylum level. (**b**) Shannon entropy. No statistically significant difference in α-diversity was observed between Groups A and B (*p* = 0.802). β-diversity based on unweighted UniFrac distance was also not significantly different between the groups according to PERMANOVA (pseudo-F = 0.794, *p* = 0.873). (**c**) Exploratory LEfSe analysis. Taxa with absolute LDA scores ≥ 2 are shown as candidate taxa contributing to intergroup differences. Taxa assigned to the genera Bacteroides and Caulobacter showed higher relative abundance in Group B in this exploratory analysis. These exploratory LEfSe findings should be interpreted as hypothesis-generating and not as confirmatory evidence of statistically significant differential abundance.

Figure 2a shows the bacterial composition of each nasal sample, and Figure 2b shows Shannon entropy. No statistically significant differences were observed in α-diversity between Groups A and B, as assessed by Shannon entropy (*p* = 0.802).

Similarly, β-diversity analysis based on unweighted UniFrac distances did not show a statistically significant difference between the groups according to PERMANOVA (pseudo-F = 0.794, *p* = 0.873).

Because overall α- and β-diversity did not differ significantly, LEfSe was used only as an exploratory, hypothesis-generating analysis to identify candidate taxa that may contribute to group-level compositional patterns. In this exploratory LEfSe analysis, 14 taxa showed LDA scores with an absolute value of ≥2. Among these, taxa assigned to the genera Bacteroides and Caulobacter showed higher relative abundance in Group B. These findings should be interpreted cautiously because they were not supported by significant differences in overall diversity metrics, antimicrobial susceptibility testing, or resistance-gene analysis of the taxa, and were not confirmed by a multiple-testing-corrected differential abundance analysis.

### 2.4. Quinolone Resistance-Determining Region (QRDR) Mutation Rates in S. epidermidis Isolated from the Conjunctival Sac and Nasal Cavity

**Figure 3:**
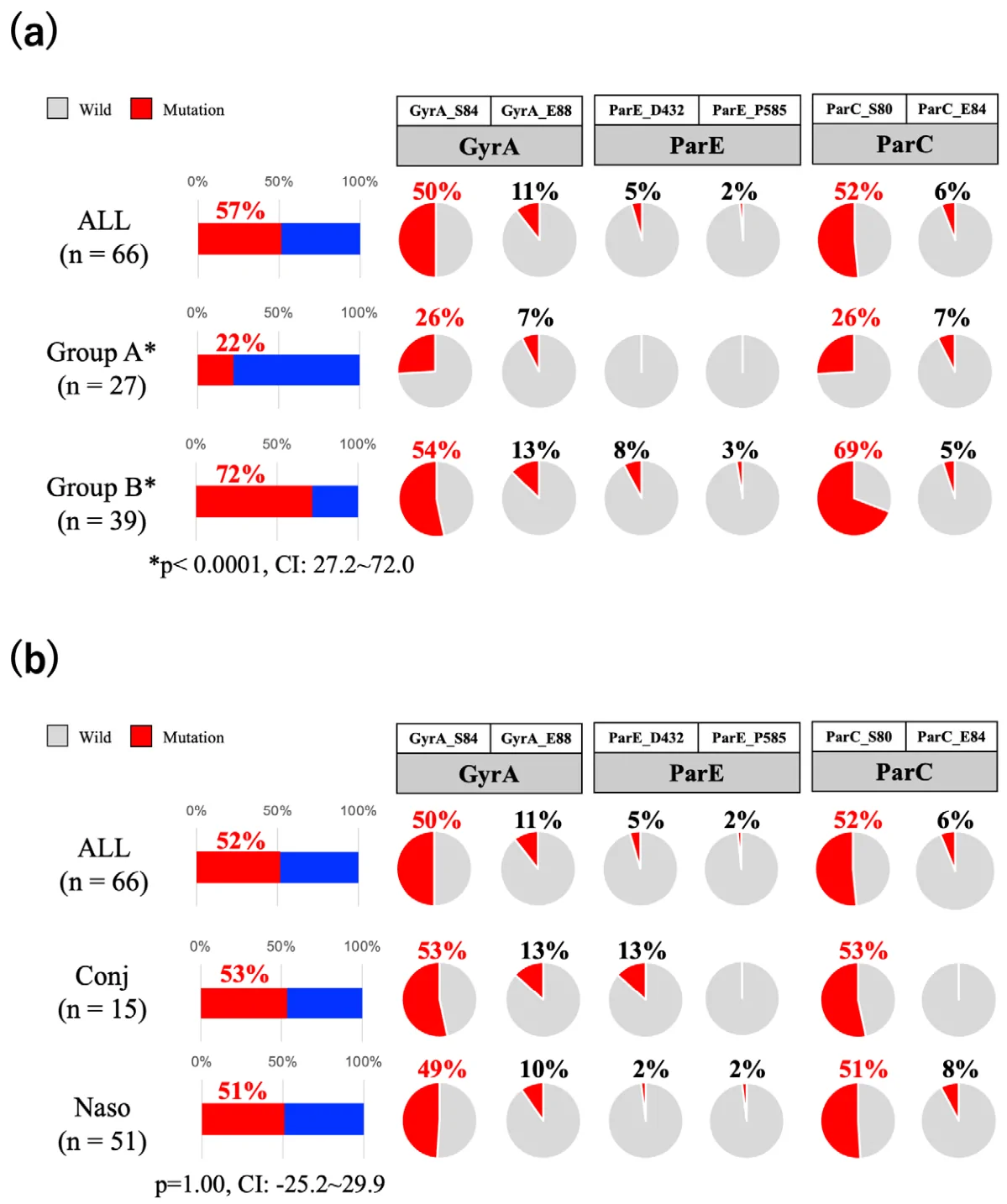
Mutation rates of QRDR-associated genes in *S. epidermidis* isolated from the conjunctival sac and nasal cavity. (**a**) Increased mutation rates were notable at S84 in the GyrA gene and S80 in the ParC gene. (**b**) When conjunctival and nasal isolates were compared, the frequency of resistance-associated gene mutations was similar.

Furthermore, all *S. epidermidis* strains isolated from the conjunctival sac and nasal cavity were analyzed for mutation rates at residues S84 and E88 of the GyrA gene, residues S80 and E84 of the ParC gene, and residues D432 and P585 of the ParE gene by classifying the strains into Group A and Group B [Figure 3a].

The most common mutations observed were GyrA_S84 and ParC_S80: GyrA_S84 (Group A, 26%; Group B, 54%) (*p* = 0.042, CI: 0.111~0.893) and ParC_S80 (Group A, 26%; Group B, 69%) (*p* = 0.0010, CI: 0.057~0.458). The overall distribution of the six mutations was 22% in Group A and 72% in Group B (*p* < 0.0001, CI: 27.2~72.0), indicating a higher frequency of amino acid mutations in the QRDR in Group B. When mutation prevalence was compared by site, as shown in Figure 3b, the S84 mutation in the GyrA gene was present in 53% of conjunctival isolates and 49% of nasal isolates, whereas the S80 mutation in the ParC gene was present in 53% of conjunctival isolates and 51% of nasal isolates (*p* = 1.00, CI: −25.2~29.9). Overall, the prevalence of QRDR mutations was 53% among conjunctival isolates and 51% among nasal isolates.

### 2.5. Phylogenetic Tree Analysis of Quinolone-Resistant S. epidermidis

**Figure 4:**
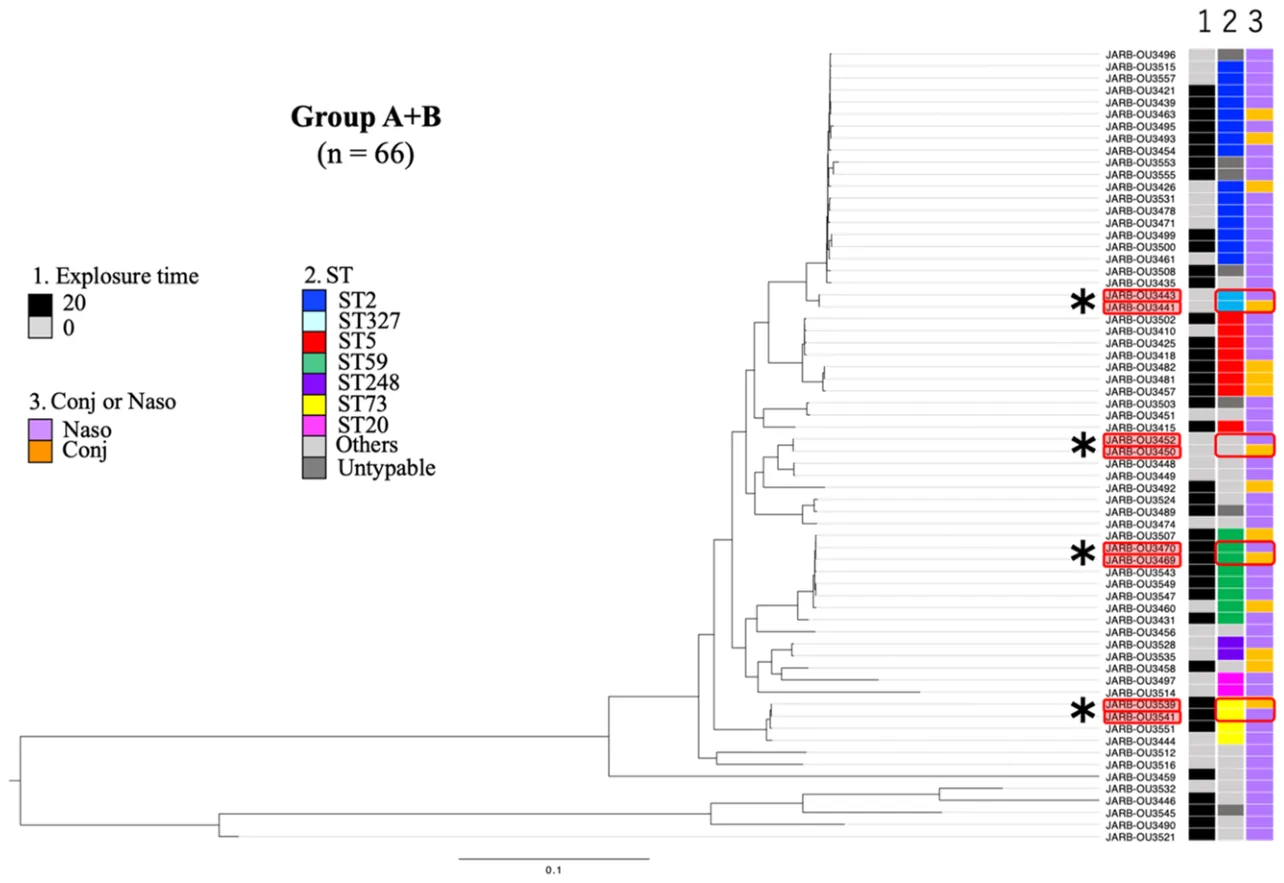
Phylogenetic tree of quinolone-resistant *S. epidermidis* isolated from all specimens. Paired isolates from the conjunctiva and nasal cavity showed the same ST type in 4 out of 10 cases (*).

To determine whether quinolone-resistant bacteria detected in the conjunctiva of each individual were the strain associated with those detected in the nasal cavity, we performed phylogenetic tree analysis on 66 strains of quinolone-resistant *S. epidermidis* isolated from both sites (Figure 4). Identity was assessed using ST typing as an indicator.

Paired conjunctival and nasal isolates were of the same ST type in 4 of 10 cases. Pairwise SNP analysis showed that the four cases had fewer than 20 SNP differences (<20 SNPs) (Supplementary Table S4).

## 3. Discussion

This study showed that repeated use of antimicrobial eye drops during anti-VEGF intravitreal injections was associated with reduced antimicrobial susceptibility in both the conjunctival sac and nasal cavity. Previous studies have also reported the effects of perioperative antimicrobial eye drops on the conjunctival microbiome following anti-VEGF intravitreal injections [4,5,6,8,10]; however, this study and our previously published work [9] investigated a substantially larger number of repeated exposures—more than 20 administrations. Although changes in the conjunctival sac microbiota associated with antibiotic eye drops have been reported using metagenomic analysis by Hotta F [1] and Kang Y [11], we demonstrated that this might also be associated with decreased antimicrobial susceptibility in the nasal cavity. In this study, the number of repeated anti-VEGF intravitreal injections ranged from 21 to 96, with a mean of 43.6 ± 18.6. Considering that some patients in clinical practice undergo even more repeated administrations and that the number of such patients is increasing, the prevalence of bacteria with reduced antimicrobial susceptibility may be even greater.

*Corynebacterium* spp. exhibited reduced susceptibility in Group B, particularly among nasal isolates. For conjunctival isolates, confidence intervals were wide and included zero; therefore, these findings should be interpreted with caution.

Among conjunctival *Corynebacterium* spp. isolates, decreases in susceptibility rates and increases in model-based mean MICs were observed from Group A to Group B; however, none of these differences reached statistical significance. Therefore, it is difficult to conclude that antimicrobial resistance increased in Group B. This may partly reflect sampling limitations, such as the extremely small volume of specimens that can be obtained from the conjunctival sac and the lower number of isolated bacteria compared to nasal swab specimens. Furthermore, we believe that the fact that MIC values were already relatively high in the conjunctival isolates from Group A may also have contributed to this finding.

In contrast, nasal *Corynebacterium* spp. isolates in Group A exhibited high susceptibility rates (88.2–100.0%) and low MIC values. The discrepancy between conjunctival and nasal isolates in Group A may reflect differences in previous antimicrobial exposure at these anatomical sites.

For nasal isolates, all three quinolones showed lower susceptibility rates and higher mean MIC values in Group B than in Group A, and these changes were more pronounced than those observed among conjunctival isolates. Although the differences did not reach statistical significance and caution is warranted when interpreting these findings as evidence of antimicrobial resistance selection induced by repeated antimicrobial eye drop administration, the observed trend toward reduced susceptibility among nasal isolates is noteworthy. Given that nasal specimens generally yield larger numbers of bacteria than conjunctival specimens, these findings may be relevant when considering the potential impact of repeated quinolone eye drop exposure on antimicrobial susceptibility.

While decreased drug susceptibility of the conjunctival microbiota due to perioperative prophylactic antibiotic administration during cataract surgery has been widely discussed, cataract surgery is typically performed only twice in a lifetime. In contrast, anti-VEGF intravitreal injections can constitute long-term, repeated treatment, leading to increased exposure to antimicrobial eye drops. These findings suggest that antimicrobial eye drops may reduce antimicrobial susceptibility even with intermittent repeated use, reaffirming the importance of this issue.

Because validated quinolone breakpoints for *C. acnes* were unavailable, the susceptibility categorizations should be interpreted cautiously. Therefore, the findings for *C. acnes* were interpreted primarily on the basis of MIC distributions and changes in susceptibility rather than categorical resistance rates. However, in dermatology, frequent use of antimicrobial agents has also been reported to induce resistance in *C. acnes* [12].

This difference may be attributable to the fact that, in daily clinical practice, treatment for *C. acnes* is often performed in dermatology, where tetracyclines and macrolides are used. Resistance to these agents is recognized as a concern, and exposure to quinolone antibiotics has been noted to be relatively rare [13,14]. A study on *C. acnes* [15] reported that although mutations in GyrA—one of the DNA gyrase subunits involved in quinolone resistance—were detected, no ParC mutant strains were identified. Although the mechanism by which *C. acnes* does not acquire ParC mutations remains unclear, these findings are noteworthy from the perspective of resistance prevention.

Regarding QRDR mutation sites associated with quinolone resistance, higher mutation rates were observed at GyrA_S84 and ParC_S80, which are considered major mutation sites; however, no new mutation sites were identified. This suggests that frequent use of antibiotic eye drops may contribute to the selection of quinolone-resistant bacteria.

Of the 10 patients with quinolone-resistant *S. epidermidis* isolated from both sites, in 4 patients, conjunctival and nasal isolates were of the same ST type. This finding suggests the possibility of genetic relatedness between isolates from the two anatomical sites, and pairwise SNP analysis showed that the four cases had <20 SNP differences [16], suggesting that the isolates belonged to the same lineage and were genetically related (Supplementary Table S4). In the other six cases, different ST types were seen and the mechanism responsible for nasal colonization by resistant isolates is unclear.

There is a report indicating a correlation between resistant isolates in the conjunctival sac and the nasal cavity [17]. Approximately 40% of topically administered eye drops are absorbed through the nasal mucosa [18]; therefore, ophthalmologists prescribing these agents should be fully aware that they may directly affect the nasal microbiota.

In the nasal microbiome analysis, no statistically significant differences were detected in α-diversity or β-diversity between Groups A and B. Therefore, the exploratory LEfSe results should be interpreted as exploratory and hypothesis-generating rather than confirmatory evidence of differential abundance. The exploratory LEfSe analysis identified several candidate taxa, including taxa assigned to the genera Bacteroides and Caulobacter, with higher relative abundance in Group B. As these microbiome data lack antimicrobial susceptibility testing or resistance-gene analysis of the taxa identified by LEfSe, the clinical significance of these compositional differences is unclear. Future studies with larger sample sizes and prespecified, multiple-testing-corrected analyses are needed to validate these candidate taxa.

This study has some limitations. The antimicrobial susceptibility analyses and the prevalence of QRDR mutations presented in this study were performed at the isolate level, and therefore may have been influenced by within-patient clustering. However, when a patient-level sensitivity analysis was performed using the isolate with the lowest isolate identification number as the representative isolate for each patient, the findings obtained from the isolate-level analysis were largely maintained, and the direction of the observed effects was consistent with that of the primary analysis. The data supporting this comparison are provided in Supplementary Table S2. In addition to the limited number of cases, patients with a history of surgery were not excluded. However, we confirmed that no surgery had been performed within the 6 months immediately before specimen collection. Additional limitations include the observational comparison of different patient groups rather than longitudinal follow-up of the same individuals, possible confounding by prior ocular surgery, systemic antibiotic exposure, hospitalization, ocular comorbidities, and topical medication use, and possible non-independence of multiple isolates obtained from the same patient. Susceptibility analyses of conjunctival *S. epidermidis* isolates were based on a limited number of non-MRSE isolates, which may have resulted in unstable susceptibility estimates. Therefore, the results should be interpreted with caution, and future studies using patient-level analyses with larger sample sizes are warranted. In microbiome analysis, no mock community control was included. Negative extraction and PCR no-template controls were not included.

Regarding the incidence of infectious endophthalmitis following intravitreal anti-VEGF injections, it has been reported that there is no difference between cases in which prophylactic antibiotic eye drops were used and those in which they were not [19,20,21,22]. Furthermore, many studies have questioned the efficacy of prophylactic antibiotic eye drops [23,24,25]. Conversely, it has also been reported that antibiotic use increases the risk of endophthalmitis by 1.54-fold [26]; proposed reasons include changes in the bacterial flora and selective induction of resistance by antibiotic eye drops [11,19,25,27,28,29].

In the United States, the American Academy of Ophthalmology recommended discontinuing routine use of topical antibiotic eye drops after intravitreal injections of anti-VEGF agents in 2013 [30]. Data from 2013 also indicated that 78% of clinicians were not using them [28]. Similar recommendations have also been issued by the Vision Academy and the American Society of Retina Specialists.

In France, the rate of pre- and postoperative prophylactic antibiotic eye drop use in conjunction with intravitreal injections of anti-VEGF agents decreased sharply from 84.6% in 2009 to 27.4% in 2018 [31]. In Japan, as of April 2024, the Japanese Society of Retina and Vitreous has also published guidelines regarding vitreous injections for macular diseases, stating that “for typical patients (those without a high risk of infection), the use of antimicrobial eye drops before and after injection is unnecessary,” and discontinuation of such use is progressing [32].

Thus, although awareness of the appropriate use of antimicrobial eye drops in conjunction with intravitreal injections of anti-VEGF agents appears to be increasing, many instances remain in daily clinical practice in which such eye drops are used too frequently and without clear justification.

We believe that these findings serve as a reminder to exercise caution in a range of clinical situations involving antimicrobial use.

## 4. Materials and Methods

This study included patients scheduled to receive anti-VEGF therapy (no exposure to topical antibiotics) or patients who had already received such therapy more than 20 times at the Department of Ophthalmology, Kansai Medical University Hospital, between 21 January 2022, and 13 January 2023. The patients were divided into two groups: 21 patients before their first use of prophylactic antimicrobial eye drops (Group A; mean age, 76.3 years) and 26 patients who had received 20 or more injections (Group B; mean age, 79.28 years). During each intravitreal injection cycle, patients in Group B received prophylactic moxifloxacin eye drops for a total of 7 days, consisting of the 3 days before the injection, the day of injection, and the 3 days after the injection. This 7-day prophylactic regimen was applied to every injection cycle, including the period before and after the final intravitreal injection. After completing the 7-day prophylactic moxifloxacin regimen administered before and after the final intravitreal injection, patients in Group B entered an observation period during which no antimicrobial eye drops were administered until specimen collection. Specimens from Group A were collected from the conjunctival sac and nasal cavity on the day intravitreal injection treatment was deemed necessary, whereas specimens from Group B were collected on scheduled follow-up visits after the final injection. Specimen collection was performed upon obtaining informed consent during the study period rather than at a fixed time point in the injection cycle. Consequently, the interval between the final administration of prophylactic moxifloxacin and specimen collection varied among patients. No antimicrobial eye drops were administered before or after specimen collection in either group. The sampling timing was not strictly standardized among patients. Specimens were collected using sterile swabs, immersed in the transport medium “ANAPort, BIKEN^®^”, stored at −80 °C, and sent to the Institute of Microbiology at Osaka University for bacteriological examination.

Aerobic culture was performed on Columbia medium supplemented with 5% fetal bovine serum for 24–48 h at 36.5 °C, and anaerobic culture was performed on chocolate agar for 24–120 h at 36.5 °C. Furthermore, the isolated bacteria were transferred to thioglycolic acid medium and cultured at 36.5 °C for 1–2 weeks for further bacterial isolation. Bacterial isolates were identified using the VITEK 2 system (bioMérieux, Marcy-l’Étoile, France) based on biochemical colorimetric reactions. *Corynebacterium* isolates were identified at the genus level only, and species-level identification was not performed.

When multiple isolates were obtained from the same patient, the same bacterial species, and the same anatomical sampling site, all isolates were included in the primary (isolate-level) analysis. To assess the potential impact of within-patient clustering, a patient-level sensitivity analysis was additionally performed, and the corresponding results are presented in Supplementary Table S2. For the patient-level analysis, the representative isolate was defined as the isolate with the lowest isolate identification number for each patient, species, and sampling site.

In this study, written informed consent was obtained from all participating patients using a consent form compliant with the Declaration of Helsinki. The study was approved by the Kansai Medical University Hospital Ethics Review Committee (approval no. 2021254).

### 4.1. Susceptibility Testing

Bacterial identification was performed using the VITEK 2 system (bioMérieux, Marcy-l’Étoile, France) based on biochemical colorimetric reactions. *Corynebacterium* isolates were identified at the genus level only, and species-level identification was not performed.

Antimicrobial susceptibility of the identified bacteria (*Staphylococcus epidermidis*, *Corynebacterium* spp., and *Cutibacterium acnes*) was determined using the broth microdilution method. Minimum inhibitory concentrations (MICs) of cefmenoxime (CMX), ceftazidime (CAZ), levofloxacin (LVFX), gatifloxacin (GFLX), and moxifloxacin (MFLX) were measured.

Susceptibility testing was performed using commercially prepared dry plates (Eiken Chemical Co., Ltd., Tokyo, Japan), according to the manufacturer’s instructions. The final inoculum size was approximately 5 × 10^4^ CFU/well for *S. epidermidis* and *Corynebacterium* spp., and 1 × 10^5^ CFU/well for *C. acnes*. *S. epidermidis* was incubated aerobically at 35 ± 2 °C for 16–20 h, whereas *Corynebacterium* spp. was incubated aerobically at 35 °C for 24–48 h. *C. acnes* was incubated anaerobically at 36 ± 1 °C for 46–48 h.

Interpretation of susceptibility results for coagulase-negative staphylococci was performed according to the Clinical and Laboratory Standards Institute (CLSI) M100-S22 guidelines [33]. For *Corynebacterium* spp., susceptibility was interpreted according to CLSI M45, 3rd edition, when applicable. For *C. acnes*, interpretive criteria for anaerobic bacteria described in CLSI M100-S22 were used as a reference. Detailed information regarding the source of interpretive criteria, guideline version, MIC breakpoints, incubation conditions, interpretive categories, and quality control strains for each organism–antimicrobial combination is provided in Supplementary Table S3.

Quality control was performed using *Escherichia coli* ATCC 25922, *Staphylococcus aureus* ATCC 29213, *Pseudomonas aeruginosa* ATCC 27853, and *Enterococcus faecalis* ATCC 29212. MIC measurements were performed once for each isolate according to routine clinical laboratory procedures.

For MIC analyses, values were log-transformed and back-transformed to the original scale, and mean MICs were compared using a linear model. Antimicrobial susceptibility was evaluated by dichotomizing isolates as susceptible (S) or non-susceptible, with intermediate (I) and resistant (R) categories combined as non-susceptible. The susceptibility rate and its 95% confidence interval for each group were caslculated using the Clopper–Pearson exact method. Between-group comparisons were performed using Fisher’s exact test (two-sided). All statistical analyses were conducted using SPSS Statistics version 26 (IBM Corp., Armonk, NY, USA).

### 4.2. Analysis of the Nasal Microbiome

DNA was extracted from residual material in ANAPort, BIKEN^®^ obtained from nasal swab samples using Maxwell^®^ (Promega Co., Ltd., Madison, WI, USA). Bacterial community composition was analyzed by sequencing the V3–V4 variable regions of the 16S rRNA gene, following the “16S Metagenomic Sequencing Library Preparation” protocol (Part # 15044223 Rev. B; Illumina, San Diego, CA, USA).

Briefly, the genomic DNA was subjected to a first-round PCR (Amplicon PCR) to amplify the V3–V4 region using locus-specific primers containing Illumina overhang adapter sequences (Forward: 5′-TCGTCGGCAGCGTCAGATGTGTATAAGAGACAGCCTACGGGNGGCWGCAG-3′; Reverse: 5′-GTCTCGTGGGCTCGGAGATGTGTATAAGAGACAGGACTACHVGGGTATCTAATCC-3′). The PCR amplification was performed under the following thermal cycling conditions: initial denaturation at 95 °C for 3 min; 25 cycles of 95 °C for 30 s, 55 °C for 30 s, and 72 °C for 30 s; and a final extension at 72 °C for 5 min.

The resulting amplicons were purified using Agencourt AMPure XP beads (Beckman Coulter, Brea, CA, USA). Subsequently, a second-round PCR (Index PCR) was performed to attach dual indices and Illumina sequencing adapters using the Nextera XT Index Kit (Illumina). The cycling conditions were as follows: 95 °C for 3 min; eight cycles of 95 °C for 30 s, 55 °C for 30 s, and 72 °C for 30 s; and a final extension at 72 °C for 5 min. The final libraries were purified again with AMPure XP beads and pooled in equimolar concentrations. Sequencing was performed on an Illumina MiSeq platform with 2 × 300 cycles, according to the manufacturer’s instructions.

The mean sequencing depth was 219,244 ± 29,894 reads per sample, with a median of 216,715 reads per sample and a range of 130,928–312,619. Samples with fewer than 80,000 reads after quality filtering were excluded from downstream analysis. Raw reads were processed using QIIME2 version 2022.2.1. Briefly, reads were demultiplexed, quality-filtered, denoised, and merged using DADA2 with the following truncation parameters: --p-trim-left-f 10 --p-trim-left-r 10 --p-trunc-len-f 280 --p-trunc-len-r 220. Amplicon sequence variants were used for downstream analysis.

Taxonomic classification was performed using a naïve Bayes classifier (silva-138-99-515-806-nb-classifier) trained on the SILVA 138 database trimmed to the amplified 16S rRNA target region. For diversity analyses, the feature table was normalized by rarefaction to 80,000 reads per sample. α-diversity was evaluated using Shannon entropy. β-diversity was evaluated using unweighted UniFrac distances, and differences between Groups A and B were tested using PERMANOVA with 10,000 permutations in QIIME2. The tested grouping variable was treatment group, and no stratification was applied.

LEfSe analysis was performed as an exploratory, hypothesis-generating analysis to identify candidate taxa contributing to intergroup differences. Relative abundance data were analyzed at the all available taxonomic levels using LEfSe version 1.1.2. The Kruskal–Wallis test threshold was set at α = 0.05, and the LDA score threshold was set at |log10 LDA| ≥ 2.0. Because LEfSe was used without a formal multiple-testing-corrected confirmatory differential abundance model, the exploratory LEfSe results were interpreted as exploratory only.

### 4.3. Variants in the QRDR of S. epidermidis

In Groups A and B, DNA was extracted from *S. epidermidis* strains isolated from conjunctival sac and nasal cavity samples using the Maxwell^®^ RSC Genomic DNA Kit. We investigated known genetic variants in the quinolone resistance-determining region (QRDR), specifically GyrA (S84, E88), ParE (D432, P585), and ParC (S80, E84). We compared the mutation rates at each site within the QRDR between Groups A and B, as well as between the conjunctival sac and the nasal cavity.

### 4.4. Phylogenetic Analysis of Quinolone-Resistant Staphylococcus epidermidis

In individuals in whom quinolone-resistant *S. epidermidis* was detected in both the conjunctiva and nasal cavity, phylogenetic analysis was performed to confirm whether the quinolone-resistant strains in the conjunctiva and nasal cavity were identical. Whole-genome sequencing was performed using the Illumina HiSeq X Five next-generation sequencer. shovill v1.0.9 was used for data assembly, and kSNP 3.0 was used for SNP calling. Subsequently, recombination regions were removed using Gubbins v3.2.0, a maximum-likelihood phylogeny was generated using RAxML-NG v1.0.1, and the results were visualized using FigTree v1.4.4. Using pairwise SNP analysis (Supplementary Table S4), pairs with fewer than 20 SNP differences (<20 SNPs) [16] were considered to belong to the same lineage and to be genetically related.

## 5. Conclusions

Repeated exposure to prophylactic antimicrobial eye drops was associated with reduced susceptibility in selected conjunctival and nasal commensal bacteria. These results support antimicrobial stewardship concerns about the routine or repeated use of topical antimicrobials in intravitreal injection therapy.

## Data Availability

Raw 16S rRNA sequencing data generated in this study have been deposited in the DNA Data Bank of Japan (DDBJ) under the accession numbers (BioProject PRJDB42423, DRA accession numbers DRR1057467–DRR1057511). Whole-genome sequencing data of *Staphylococcus epidermidis* obtained in this study have been deposited in the DNA Data Bank of Japan under the accession numbers (BioProject PRJDB42388, DRA accession numbers DRR1056055–DRR1056120). MIC, QRDR isolate metadata, SNP matrices, and phylogenetic tree files are available as Supplementary Tables S4–S7.

## Supplementary Materials

The following supporting information can be downloaded at: https://www.mdpi.com/article/10.3390/antibiotics15080744/s1, **Figure S1:** Changes in the mean MIC value (µg/mL) of various antibiotics against *S. epidermidis.* **Table S1:** Changes in the susceptibility of *S. epidermidis* to MFLX following frequent administration of antimicrobial eye drops (isolate-level). These data were previously published in [9] and are reproduced here for comparison. **Table S2:** Patient-level analysis of Table S2a MICdata (Patient-level), Table S2b QRDRdata (Patient-level), antimicrobial susceptibility rates of *Corynebacterium* spp. (corresponding to Table S2c–e), antimicrobial susceptibility rates of *Staphylococcus epidermidis* (corresponding to Table S2f), and QRDR mutation rates (corresponding to Figure S2a,b). **Table S3:** Susceptibility-testing methods. Sources of interpretive criteria, guideline versions/years, MIC cutoff, incubation conditions, interpretive categories, and quality control strains used for antimicrobial susceptibility testing. **Table S4:** Pairwise SNP distance matrix of *Staphylococcus epidermidis* isolates included in the phylogenetic analysis. **Table S5:** Metadata and assembly metrics of *Staphylococcus epidermidis* isolates recovered from the nasal cavity and analyzed by whole-genome sequencing. **Table S6:** Raw minimum inhibitory concentration (MIC) data for all bacterial isolates included in this study. **Table S7:** Branch support values of phylogenetic tree.

## Abbreviations

The following abbreviations are used in this manuscript:

anti-VEGF	anti-vascular endothelial growth factor
MIC	minimum inhibitory concentration
CMX	Cefmenoxime
CAZ	Ceftazidime
LVFX	Levofloxacin
GFLX	Gatifloxacin
MFLX	Moxifloxacin
QRDR	quinolone resistance-determining region
*S. epidermidis*	*Staphylococcus epidermidis*
*C. acnes*	*Cutibacterium acnes*
TGC	thioglycolic acid
DNA	deoxyribonucleic acid
rRNA	ribosomal ribonucleic acid
PCR	polymerase chain reaction
QIIME2	Quantitative Insights Into Microbial Ecology 2
LEfSe	Linear Discriminant Analysis Effect Size
LDA	linear discriminant analysis
CI	confidence interval
SNP	single-nucleotide polymorphism
ST	sequence type
