# Supplementary material for: Association Between Repeated Prophylactic Antimicrobial Eye Drop Use and Reduced Antimicrobial Susceptibility in Conjunctival and Nasal Commensal Bacteria
Source: Antibiotics (Basel). 2026 Jul 31;15(8):744. doi: 10.3390/antibiotics15080744 (PMC13509217; doi:10.3390/antibiotics15080744)
Supplement: Supplementary file 1 [file antibiotics-15-00744-s001.zip › Table S7.pdf]

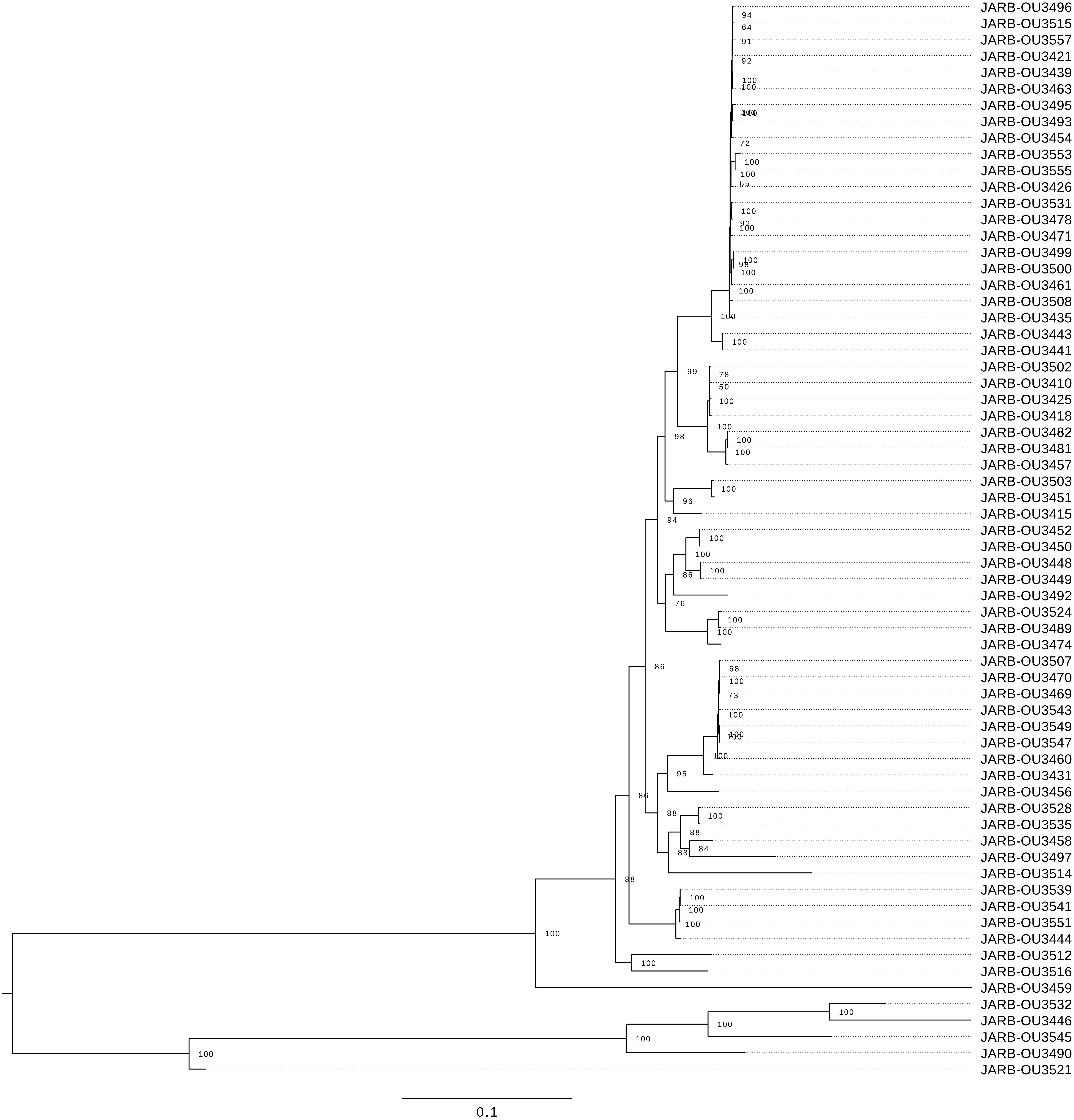
JARB-OU3496
JARB-OU3515
JARB-OU3557
JARB-OU3421
JARB-OU3439
JARB-OU3463
JARB-OU3495
JARB-OU3493
JARB-OU3454
JARB-OU3553
JARB-OU3555
JARB-OU3426
JARB-OU3531
JARB-OU3478
JARB-OU3471
JARB-OU3499
JARB-OU3500
JARB-OU3461
JARB-OU3508
JARB-OU3435
JARB-OU3443
JARB-OU3441
JARB-OU3502
JARB-OU3410
JARB-OU3425
JARB-OU3418
JARB-OU3482
JARB-OU3481
JARB-OU3457
JARB-OU3503
JARB-OU3451
JARB-OU3415
JARB-OU3452
JARB-OU3450
JARB-OU3448
JARB-OU3449
JARB-OU3492
JARB-OU3524
JARB-OU3489
JARB-OU3474
JARB-OU3507
JARB-OU3470
JARB-OU3469
JARB-OU3543
JARB-OU3549
JARB-OU3547
JARB-OU3460
JARB-OU3431
JARB-OU3456
JARB-OU3528
JARB-OU3535
JARB-OU3458
JARB-OU3497
JARB-OU3514
JARB-OU3539
JARB-OU3541
JARB-OU3551
JARB-OU3444
JARB-OU3512
JARB-OU3516
JARB-OU3459
JARB-OU3532
JARB-OU3446
JARB-OU3545
JARB-OU3490
JARB-OU3521
0.1
